# Dynamics of metal binding and mutation in yybP–ykoY riboswitch of *Lactococcus lactis*

**DOI:** 10.1039/d2ra02189g

**Published:** 2022-06-13

**Authors:** Mazhar Iqbal, Syed Tarique Moin

**Affiliations:** Third World Center for Science and Technology, H. E. J. Research Institute of Chemistry, International Center for Chemical and Biological Sciences, University of Karachi Karachi-75270 Pakistan tarique.syed@iccs.edu +92-21-348-19018 +92-21-99261774

## Abstract

Riboswitch is a regulatory segment of messenger RNA (mRNA), which by binding to various cellular metabolites regulates the activity of mRNA *via* modulating transcription, translation, alternative splicing, and stability of the mRNA. yybP–ykoY riboswitch of *Lactococcus lactis*, which is present upstream of the yoaB gene, functions as a Mn^2+^-specific genetic ON-switch, and modulates expression of proteins which are significant for Mn^2+^ homeostasis. The P1.1 switch helix of the aptamer domain of the riboswitch contains an intrinsic transcription terminator structure, which gets stabilized with Mn^2+^ binding and causes disruption of terminator structure and allows the continuation of transcription. The current research work involved the evaluation of structural and dynamical properties of the yybP-ykoY riboswitch of *L. lactis* in its Mn^2+^-free, Mn^2+^-bound (wild-type), and Mn^2+^-bound mutant (A41U) states by applying molecular dynamics simulations. Based on the simulations, the effects of Mn^2+^ absence and A41U mutation were evaluated on the structure and dynamics of the riboswitches followed by the computation of the free energy of metal binding in the wild-type and the mutant riboswitches. The simulation results provided insights into the properties of the riboswitch with the focus on the dynamics of the P1.1 switch helix, and the manganese binding site designated as M_B_ site, as well as the relative stability of the wild-type and the mutant riboswitches, which helped to understand the structural and dynamical role of the metal ion involved in the function of Mn^2+^-sensing riboswitch.

## Abstract 2

The current research work involved the evaluation of structural and dynamical properties of yybP–ykoY riboswitch of *L. lactis* in Mn^2+^-free, Mn^2+^-bound (wild-type), and Mn^2+^-bound mutant (A41U) states by applying molecular dynamics simulations.

## Graphical abstract

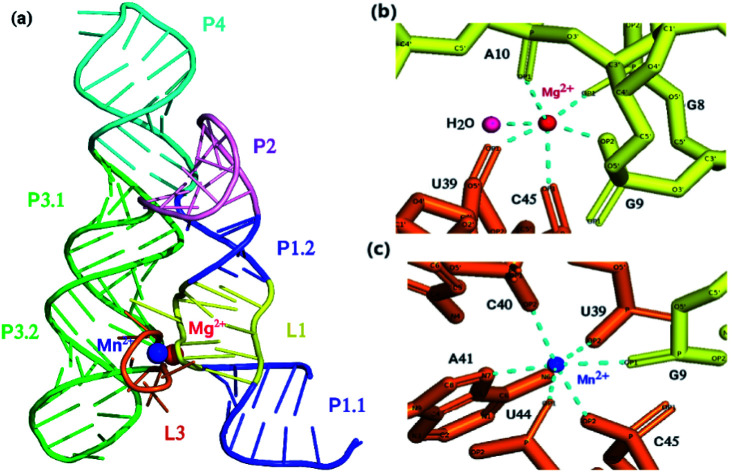

## 1 Introduction

Riboswitch is a regulatory segment of messenger RNA (mRNA) which by binding to various cellular metabolites regulates activity of mRNA by modulating transcription, translation, alternative splicing and stability of mRNA.^1,2^ To date, nearly 40 different classes of riboswitches are known which respond to different ligands along with metabolites, transfer RNAs (tRNAs), enzyme co-factors, signaling molecules and metal ions such as Mn^2+^ and Mg^2+^.^1–3^ They are largely present at 5′ untranslated regions of protein coding genes unlike thiamine pyrophosphate (TPP)-sensing riboswitch which occurs at 3′ untranslated region or in introns of target genes.^1,2,4^ Riboswitches act as genetic switch which modulate gene expression in distinct fungi, bacteria, plants, and archaea.^1,2^ Genes that are regulated by riboswitches encode proteins involved in biosynthesis, catabolism, transport and signaling of cellular metabolites that bind to the riboswitches. The regulation of gene expression by riboswitches involves either transcription termination by the formation of rho-independent transcription terminator hairpin or transcription continuation with the formation of anti-terminator structure. Likewise, gene regulation takes place either at the level of translation by sequestering the ribosome binding site to restrict translation initiation or by exposing the ribosome binding site to enable translation.^1,2^ Some riboswitches cleave themselves acting as ribozymes in the presence of ample concentration of metabolites,^5^ and some riboswitches, in eukaryotes and prokaryotes, regulate splicing of the pre-mRNA.^1,2,4,6,7^ Riboswitches contain two domains; one is aptamer (sensory domain) whose sequence is highly conserved that directly binds with metabolites, and other is expression platform whose sequence greatly varies^8^ that as a regulatory domain can either repress or activate gene expression by experiencing structural modifications in response to the changes in the aptamer structure.^9^

**Fig. 1:**
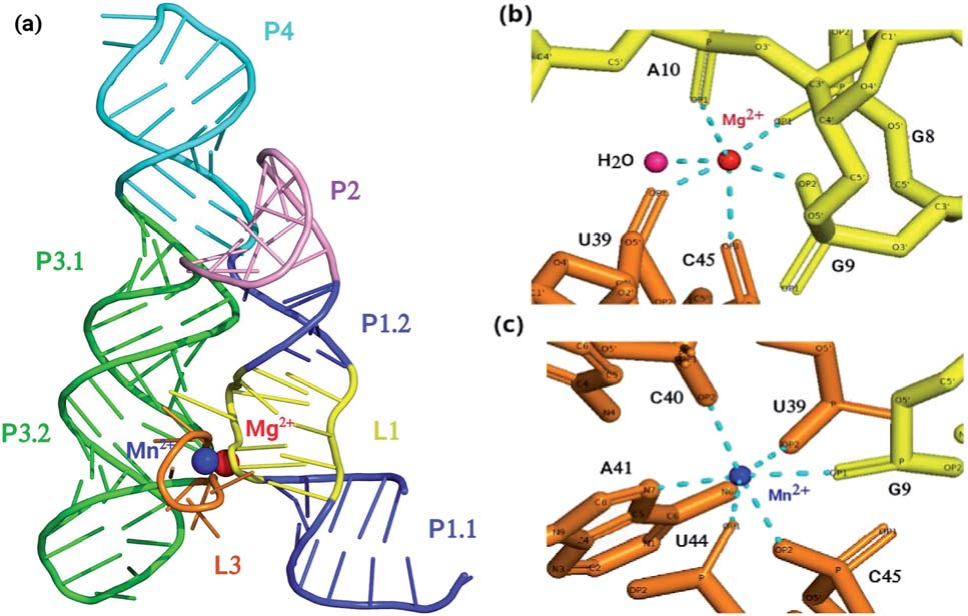
(a) Crystal structure of aptamer domain of yybP–ykoY riboswitch of L. Lactis coordinated with Mg^2+^ ion and Mn^2+^ ion. (b) Coordination scheme of Mg^2+^ ion and (c) Mn^2+^ ion. At M_A_ site, Mg^2+^ (red) is coordinated octahedrally with water and five phosphoryl oxygen atoms, whereas, at M_B_ site, Mn^2+^ (blue) is coordinated with five phosphoryl oxygen atoms and N7 of invariable adenosine (A41).

yybP-ykoY riboswitch that is mostly present in plants and human bacterial pathogens for instance *Lactococcus lactis*, *Bacillus subtilis*, *Escherichia coli*, *Xanthomonas oryzae*, *etc.*, has high sensitivity for manganese (Mn^2+^) ion. It senses charge, geometry and Lewis acid hardness of Mn^2+^ by making inner coordination sphere contacts directly with five phosphoryl oxygen atoms and one nitrogen atom prototyped as OP1_G9_, OP1_U44_, OP2_C45_, OP2_U39_, OP2_C40_, and N7_A41_ in *L. lactis*.^10^ It also regulates distinct genes and is involved in Mn^2+^ homeostasis.^10^ There are two metal binding sites designated as M_A_ and M_B_ in the riboswitch of *L. lactis*. Magnesium (Mg^2+^) ion, owing to its more abundance than Mn^2+^ at physiological conditions, and having high similarity in ionic radius, charge and octahedral coordination scheme with Mn^2+^, occupies the M_A_ site, whereas the M_B_ site has high selectivity for Mn^2+^ due to its coordination with nitrogen atom (N7) of invariable adenosine (A41). The hairpin like structure of yybP–ykoY riboswitch contains four helices which are designated as P1, P2, P3 and P4. Two super helices formed by the co-axial stacking of the four helices (P1–P4) are docked at the conserved L1 and L3 loops, and this docked interface is stabilized by two highly coordinated metal ions (Mg^2+^ and Mn^2+^) illustrated in Fig. 1. *In vitro* as well as *in vivo* studies revealed that yybP–ykoY riboswitch at upstream of yoaB gene functions as Mn^2+^-specific genetic ON-switch, and modulates expression of proteins significant for Mn^2+^ homeostasis.^11^ The P1.1 helix of the aptamer domain contains intrinsic transcription terminator. In the absence of Mn^2+^ ion, RNA polymerase constructs predominantly terminated transcript, however with Mn^2+^ binding, the aptamer domain gets stabilized and causes disruption of transcription-terminator structure and allows continuation of transcription.^11,12^

One experimental study was reported on the crystallization of *L. lactis* yybP–ykoY riboswitch in its ligand-bound form, and its comparison with that of the *E. coli* riboswitch in Mn^2+^-free form.^13^ Unlike Mn^2+^-bound crystal structure of yybP–ykoY riboswitch of *L. lactis*, the crystal structure of the Mn^2+^-free riboswitch has not been reported yet. Furthermore, to the best of our knowledge, no computational study has also been carried out which could describe complete dynamics of Mn^2+^-free riboswitch of *L. lactis*, thus leaving behind a question on how yybP–ykoY riboswitch of *L. lactis* changes its structural and dynamical properties in the absence of Mn^2+^ from the M_B_ site. Although, molecular dynamics simulations were carried out for yybP–ykoY riboswitches of *L. lactis* and *X. oryzae* in which divalent metal ions present in the metal-binding sites (M_A_ and M_B_) were replaced with monovalent K^+^ ions and their effect on the structural flexibility was determined,^14^ however, a detail study has not been reported yet, comparing the dynamics of Mn^2+^-bound yybP–ykoY riboswitch regarded as the wild-type, which contains Mg^2+^ and Mn^2+^ at the M_A_ and the M_B_ site, respectively, A41U Mn^2+^-bound – a mutant in which an invariable Mn^2+^-sensing adenosine is replaced with uracil, and Mn^2+^-free with Mg^2+^ at M_A_ site only of yybP–ykoY riboswitches of *L. lactis*. The aim of this study was to gain deep understanding of the structural and dynamical properties of the Mn^2+^-free, Mn^2+^-bound and A41U Mn^2+^-bound yybP-ykoY riboswitches of *L. lactis* at the atomistic details.

In one experimental study, single molecule kinetic analysis of RNA transient structure (SiM-KARTS) assay showed that the anti-terminating P1.1 switch helix in yybP–ykoY riboswitch gets stabilized as Mn^2+^ binds to M_B_ site *via* signal transduction through metal ion sensing core, thus restraining the formation of transcription-terminator hairpin. This stabilizing effect is lost upon mutation of the invariable Mn^2+^-sensing adenosine (A41) with uridine.^14^ Through molecular dynamics simulations, we elaborated the metal binding and the mutation effect in *L. lactis* yybP–ykoY riboswitch as well as found how the stability of P1.1 switch helix is affected in the absence of Mn^2+^. To achieve the objective, we ran 1 μs all-atom MD simulations of the Mn^2+^-free, Mn^2+^-bound and A41U Mn^2+^-bound riboswitches and came up with the detailed description of the structural and dynamical properties with the focus on the dynamic flexibility of the P1.1 switch helix.

## 2 Methods

The crystal structure of the aptamer domain of yybP–ykoY riboswitch of *L. lactis* was retrieved from protein databank (PDB ID: 4Y1I)^11^ that was an asymmetric unit containing two identical chains A and B with a total RMS of 0.58 Å.^11^ Chain A was extracted from the crystal unit, and all atoms except ribonucleotides, 11 Mg^2+^ ions, and a Mn^2+^ ion, were removed from the chain, which was taken as an initial structure that corresponded to Mn^2+^-bound riboswitch, whereas the Mn^2+^-free riboswitch was constructed by removing Mn^2+^ ion from M_B_ site in the metal bound riboswitch and the mutant form of riboswitch was obtained by introducing A41U point mutation in the metal bound riboswitch with the help of Chimera visualization program.^15^

Initial configuration of the simulation systems was obtained by using xLEaP module of AMBER20 in which ff99bsc0χOL3 AMBER force field^16–18^ was employed for modeling the ribonucleotides, and non-bonded model approach was adopted to treat the metal ions in which the metal center was described as simple Lennard-Jones sphere with an integer charge considering only electrostatic and van der Waals (vdW) terms which were used to describe the interaction between the metal ions and its surroundings. Here, the metal–ligand interactions were described through electrostatic and van der Waals parameters, with *σ* and *ε* values of 1.40 Å and 0.0168 kcal mol^−1^, and 1.36 Å and 0.0102 kcal mol^−1^, for Mn^2+^ and Mg^2+^, respectively.^19^ The implementation of the force field parameters to these three states of the riboswitch was followed by protonation, neutralization with Na^+^ as counter ions and solvation with 12 Å buffer of TIP3P water model^20^ in a truncated octahedral box with periodic boundary conditions.

### 2.1 Simulation protocol

The initial configurations of all systems (Mn^2+^-free, Mn^2+^-bound and A41U Mn^2+^-bound riboswitches) were initially subjected to energy minimization in a total 1500 steps to eliminate bad intra- and inter-molecular contacts between atoms. First, 500 steps minimization was carried out with positional restraint of 25 kcal mol^−1^ Å^−2^ on all nucleotides including all the ions except water molecules. The positional restraint was then gradually reduced by 5 kcal mol^−1^ Å^−2^ for every 100 steps followed by 500 steps unrestrained minimization. After minimization, each system was gradually heated up to ∼300 K over 80 ps under NVT (constant particle number, volume and temperature) conditions using Langevin thermostat^21^ with constraints applied to bonds involving hydrogen using SHAKE algorithm^22^ and the positional restraint with force constant of 25 kcal mol^−1^ Å^−2^ was applied to all solutes except water molecules. Each system was then equilibrated for 140 ps in NPT ensemble (*T* = ∼300 K, *P* = 1 atm) employing Langevin thermostat and Berendsen barostat.^23^ SHAKE algorithm was remained active till the production MD stage to constrain position of hydrogen atoms with force constant of 25 kcal mol^−1^ Å^−2^ applied on all atoms except water molecules. Afterward, the positional restraint was gradually released in steps with a difference of 5 kcal mol^−1^ Å^−2^. Long-range electrostatic interactions were calculated with particle mesh Ewald (PME)^24^ algorithm using cut-off distance of 12 Å. Finally, each system was subjected to production MD in the NPT ensemble using CUDA version of PMEMD module implemented in AMBER 20 suite of program for 1 μs simulation with time-step of 2 fs at ∼300 K and 1 atm set for the temperature and pressure, respectively.^25^

**Scheme 1:**
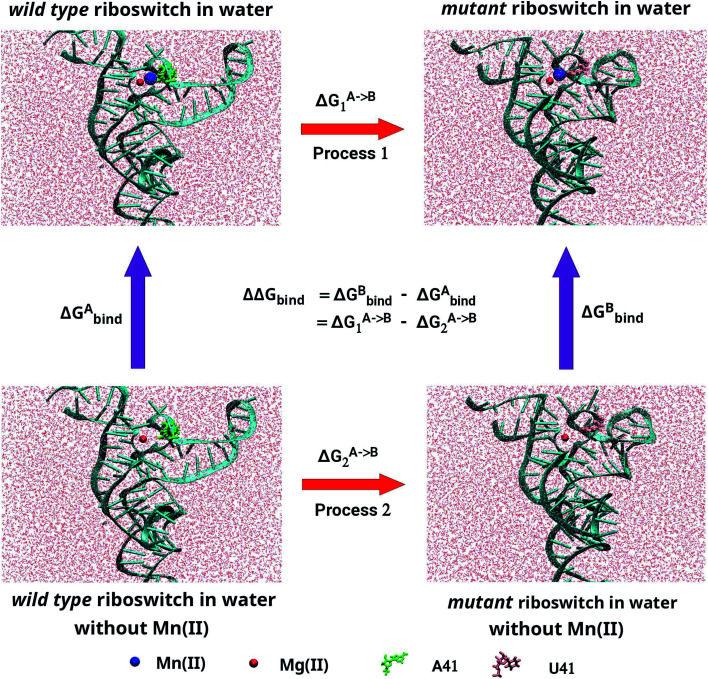
: Thermodynamic cycle employed for the computation of relative binding free energy difference between wild type and mutant yybP–ykoY riboswitches.

The relative stability of the wild-type (Mn^2+^-bound) and the mutant (A41U Mn^2+^-bound) riboswitches were estimated in terms of relative binding free energy (ΔΔ*G*_bind_) that was calculated as the free energy (Δ*G*_AB_) difference of the transformation of wild-type form into mutant (A41U) form with Mn^2+^ ion at M_B_ site in process 1 and without Mn^2+^ ion at M_B_ site in process 2 employing thermodynamic integration (TI) approach (Scheme 1). TI based free energy estimation method computes the free energy difference between the two states A and B, in this case wild-type and mutant riboswitches, by coupling them *via* a parameter *λ* that serves as an additional, non-spatial coordinate, which allows the free energy difference between the states to be computed as:1
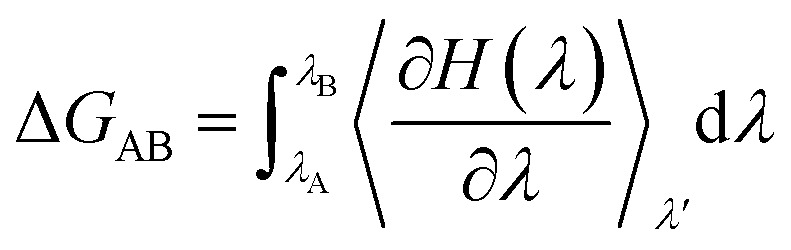


The Hamiltonian, *H* that indicates the extent of change came about between the wild-type (state A) and mutant (state B), was calculated as a function of coupling parameter *λ*. TI simulations were performed for each process employing one-step protocol that is based on disappearing one unique residue and appearing other unique residue simultaneously. Atoms of both unique residues were in softcore region and were individually specified during calculation, and both van der Waals (vdW) and charge interactions of disappearing or appearing unique atoms with neighboring atoms were illustrated by softcore potentials. We used dual topology approach in which both the wild-type and the mutant molecules were considered, simultaneously and atoms of common residues in both states were having same coordinates. To remove redundant bonding term calculations, both the wild-type and the mutant molecules were merged in such a way that the new topology contained only the common atoms and the softcore atoms which were atoms of both unique residues. TI simulations were run for each *λ* value and gradient of Hamiltonian *H* with respect to the coupling parameter, *λ*, was averaged over numerous equilibrated configurations generated during simulations that was then numerically integrated to get free energy difference (ΔΔ*G*_bind_) of the two states. In the current study, the free energy simulation protocol employed 6 *λ* points with an array of 0.2 from 0.0 to 1.0, and 1 ns TI simulation was performed for each *λ* point at the isobaric-isothermal ensemble to obtain ΔΔ*G*_bind_. The simulation systems were minimized at each *λ* point under periodic boundary conditions, employing steepest-descent algorithm prior to TI simulation. Afterward, the systems were heated up to ∼300 K over 20 ps under NVT conditions using weak-coupling algorithm with constraints applied to bonds involving hydrogen atoms using SHAKE algorithm, and positional restraint with force constant of 5 kcal mol^−1^ Å^−2^ was also applied to all atoms except water molecules and hydrogen atoms. Finally, each simulation system was simulated in the isobaric–isothermal (NPT) ensemble at each *λ* point for 1 ns with time-step of 2 fs employing CPU version of pmemd module implemented in AMBER 20;^26^ the stretching of bonds involving hydrogen atoms was constrained with SHAKE algorithm, temperature at 300 K and pressure at 1 atm were regulated by Langevin thermostat with collision frequency of 2 ps^−1^ and Berendsen barostat with pressure relaxation time of 2 ps, respectively. Softcore potential was employed, and *α* and *β* parameters of the softcore potential were set to 0.5 and 12 Å^2^, respectively. All essential Δ*G* values were obtained by numerically integrating ∂*H*/∂*λ* values collected during the free energy simulations.

The simulation trajectories were primarily analyzed in terms of root mean square deviation (RMSD) of the backbone atoms (P, O5′, C5′, C4′, C3′ & O3′) and heavy atoms belonging to nucleotides of the M_B_ site employing crystallographic structure as a reference *via* CppTraj,^27^ an analysis tool implemented in AMBERTOOLS 20. Moreover, radius of gyration (*R*_g_), root mean square fluctuation (RMSF), and dynamic cross correlation matrix (DCCM) were also evaluated. A correlation plot between *R*_g_ and RMSD was obtained with the help of pytraj, a python version of CppTraj. Essential dynamics were obtained *via* principal component analysis (PCA) using the gromacs analysis tools.^28–32^ The trajectories were visualized employing VMD program and the images were captured with the help of CHIMERA and PYMOL,^33^ along with VMD.^34^

## 3 Results and discussion

**Fig. 2:**
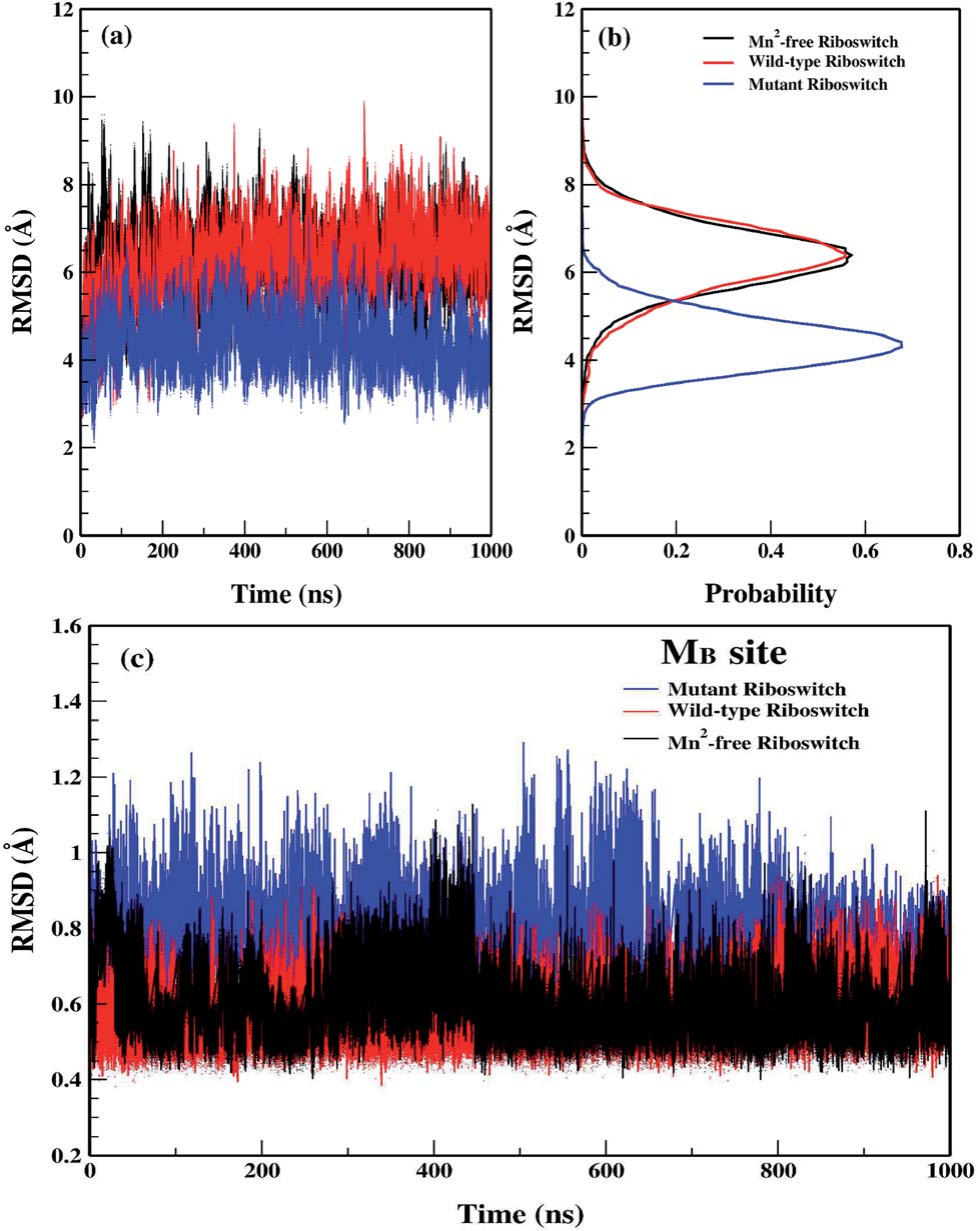
(a) Root mean square deviation (RMSD) plots of backbone atoms of Mn^2+^-free (black), wild-type (red) & mutant (blue) riboswitches as a function of simulation time. RMSD was calculated by taking crystallographic structure as a reference structure. (b) Probability plots of RMSD. (c) RMSD plots of M_B_ site residues as a function of simulation time.

The structural and dynamical properties of the Mn^2+^-free, Mn^2+^-bound (wild-type) and A41U Mn^2+^-bound (mutant) riboswitches were primarily assessed *via* time evolution root mean square deviation (RMSD) for the backbone atoms shown in Fig. 2a. For the case of Mn^2+^-free riboswitch, RMSD was stabilized after 20 ns, whereas stabilization of the RMSD occurred after 100 and 40 ns for the case of wild-type and mutant riboswitches, respectively. The averaged RMSD was computed to be approximately same for Mn^2+^-free (6.28 ± 0.78 Å) and the wild-type riboswitch (6.27 ± 0.81 Å), which were also deduced from the probability plots shown in Fig. 2b. On the other hand, the RMSD value for the mutant riboswitch was found to be lower (4.39 ± 0.60 Å) than those for the case of other two riboswitches. The very close RMSD values for the case of Mn^2+^-free and wild-type riboswitches indicated that the magnesium ions in the solution diffused to occupy the M_B_ site, thus stabilizing the RMSD profile of Mn^2+^-free riboswitch as observed in case of the wild-type. The coordination of the magnesium ion to the Mn^2+^-free riboswitch was also visualized in the simulation trajectory *via* VMD. The similar structure behaviour of Mn^2+^ and Mg^2+^ toward the riboswitch was inferred as both ions have same charge, Lewis acid hardness and octahedral coordination geometry.^35^ The RMSD profile of the mutant riboswitch was significantly lower than that of the wild-type riboswitch thus indicating higher stability of the mutant riboswitch compared to the latter one as well as reflecting that the mutation induced global structural changes to the riboswitch.

Fig. 2c illustrated the RMSD profiles of the M_B_ site nucleotides in Mn^2+^-free, wild-type and mutant riboswitches. In case of Mn^2+^-free riboswitch, the RMSD profile exhibited more fluctuation compared to the wild-type case, even though the averaged RMSD of the Mn^2+^-free riboswitch (0.59 ± 0.09 Å) was close to the wild-type case (0.56 ± 0.06 Å) but lower than that of the case of mutant riboswitch (0.78 ± 0.08 Å). The variation in RMSDs pointed toward a contrast dynamical behaviour of the two ions, however, both were reportedly having very similar structural properties.^35^ Moreover, the variation in the backbone RMSD was lower in both cases (Mn^2+^-free and wild-type riboswitches) compared to that of the mutant case thus suggesting that the overall dynamics of the M_B_ site in Mn^2+^-free riboswitch was lower than that of the mutant riboswitch, however it was conversely observed for the M_B_ site RMSD as the mutant riboswitch case demonstrated high fluctuations than that of the wild-type thus pointing toward the lower stability of the metal-binding site due to mutation. The contrasting behaviour was attributed to the mutation in the riboswitch that increased dynamic flexibility of the metal-binding site but caused the overall structure of the riboswitch less flexible. The RMSD data thus reflected that the dynamics of yybP–ykoY riboswitch was altered with the mutation and absence of manganese ion, which therefore led to further evaluate the effect of mutation and Mn^2+^ absence.

**Fig. 3:**
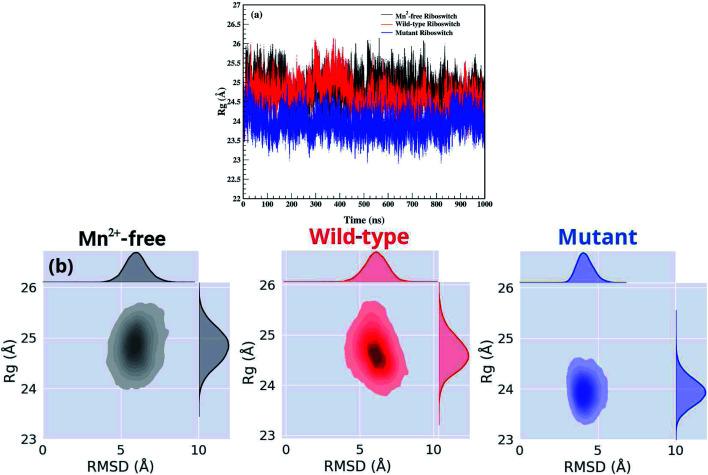
(a) Radius of gyration (*R*_g_) plots of backbone atoms of Mn^2+^-free (black), wild type (red) & mutant (blue) riboswitches as a function of simulation time. (b) Correlation plot for *R*_g_*vs.* RMSD of backbone atoms of Mn^2+^-free, wild-type & mutant yybP–ykoY riboswitches.

Time evolution radius of gyration (*R*_g_) of the backbone atoms of the three riboswitches was computed to obtain information of the overall dynamics in the absence and presence of the metal ion, and due to the mutation shown in Fig. 3a. *R*_g_ gives estimation of a system's compactness as the higher *R*_g_ value indicates lower compactness.^36^ The average *R*_g_ value for the Mn^2+^-free riboswitch (24.82 ± 0.35 Å) was very close to that of the wild-type (24.64 ± 0.35 Å) but higher than the mutant riboswitch (23.94 ± 0.27 Å), which indicated that the volume of the Mn^2+^-free and the wild-type riboswitches was indifferent demonstrating that Mn^2+^ absence has little effect on the overall structure of the riboswitch, however, their volume was slightly higher compared to the mutant riboswitch. Fig. 3b displayed the *R*_g_*vs.* RMSD correlation plots; in case of Mn^2+^-free riboswitch, distribution of the sampled conformers was located at RMSD from 4.05 to 8.05 Å corresponding to *R*_g_ value between 24.0 and 25.7 Å, whereas for the wild-type case, the corresponding distribution showed similar RMSD from 4.01 to 8.29 Å and *R*_g_ from 25.69 to 23.8 Å. In the case of the mutant riboswitch, RMSD appeared between 2.99 and 5.60 Å which corresponded to *R*_g_ value from 23.29 to 24.63 Å. The correlation plots exhibited that the dynamics of the Mn^2+^-free and wild-type riboswitches yielded to be similar, whereas the mutant case demonstrated distinct dynamics than the other two riboswitches.^37^

**Fig. 4:**
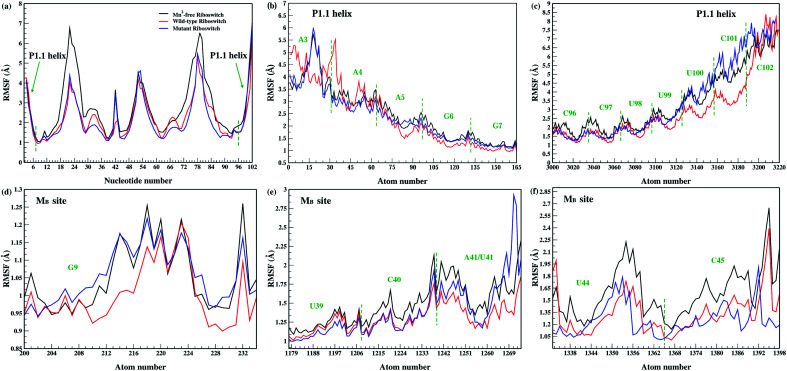
(a) RMSF around average nucleotides position of 1 μs trajectories of Mn^2+^-free (black), wild-type (red) & mutant (blue) yybP–ykoY riboswitches. (b) and (c) RMSF of P1.1 helix. (d)–(f) RMSF of M_B_ site.

Effect of Mn^2+^ absence and A41U mutation on the dynamics of the riboswitch, in particular the P1.1 switch helix, was analyzed by root mean square fluctuation (RMSF), which facilitated to understand the flexibility of different nucleotide regions during simulations.^38^Fig. 4a showed the RMSF profiles of the Mn^2+^-free, the wild-type and the mutant riboswitch; in the case of Mn^2+^-free riboswitch, residual fluctuations were higher than that of the wild-type riboswitch except for U66, G67, U68 and U86 nucleotides, and the M_B_ site nucleotides (G9, U39, C40, A41, U44 and C45) exhibited high fluctuations depicted in Fig. 4d–f. The higher flexibility of different regions in Mn^2+^-free riboswitch compared to that in the wild-type was attributed to the absence of the manganese ion which caused the riboswitch more flexible. A similar fluctuation trend was observed for the P1.1 switch helix comprising of A5, G6, G7, G8, C96, C97, U98, U99, U100 and C101 nucleotides in the Mn^2+^-free riboswitch demonstrating high flexibility as deduced from the high intensity fluctuations in the RMSF profiles shown in Fig. 4b and c. This was interpreted as manganese and magnesium ions reportedly have distinct dynamical properties therefore, the binding of Mg^2+^ ion to the Mn^2+^-free riboswitch was assumed altered thereby destabilizing the M_B_ site and the P1.1 switch helix. The stabilization of these regions can only be achieved only if the suitable metal ion like Mn^2+^ essentially binds to the metal-binding site as in the wild-type riboswitch, since according to an experimental study, the binding of Mn^2+^ ion disrupts transcription terminator hairpin structure and thus stabilizes anti-terminating P1.1 switch helix leading to transcription continuance.^11,20^ The RMSF profile of the mutant riboswitch showed mixed residual fluctuations in comparison to the wild-type case as shown in Fig. 4a, and the M_B_ site residues in particular G9 and U41 nucleotides showed higher fluctuations, and U39 and C40 nucleotides showed lower fluctuations, whereas U44 and C45 nucleotides possessed similar fluctuations as observed in case of wild-type riboswitch (Fig. 4d–f). Nucleotides of the P1.1 switch helix, in the mutant riboswitch showed distinct fluctuation pattern compared to the wild-type, since A3 and A4 nucleotides produced low fluctuations compared to G6, G7, U100, C101 and C102 nucleotides having high fluctuations, whereas A5, C96 C97 U98 and U99 nucleotides possessed similar fluctuations like those of the wild-type riboswitch (Fig. 4b and c). This implied that the mutation had mixed effect on the overall structure flexibility of the riboswitch, including the M_B_ site and the P1.1 helix.

**Fig. 5:**
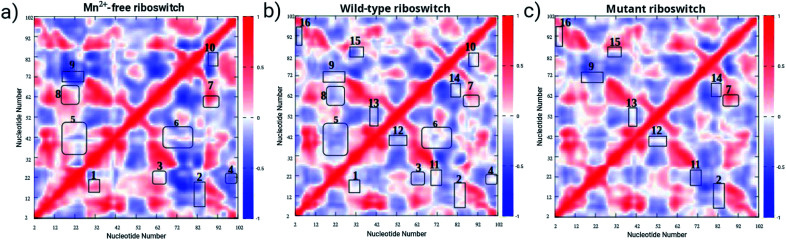
Dynamic cross correlation matrix (DCCM) of backbone atoms of (a) Mn^2+^-free, (b) wild-type, (c) and mutant yybP–ykoY riboswitches. DCCM maps, obtained from 1 μs trajectories, contain contours of different colors. Correlated motion and anti-correlated motions are represented by red and dark-blue color contours, respectively.

It was therefore observed that the mutation perturbed the M_B_ site despite an overall structural stabilization occurred in the mutant riboswitch. To evaluate the contrast in the structure and dynamics of the wild-type riboswitch in comparison to other two riboswitches, comparative dynamic cross-correlation matrix (DCCM) analysis was carried out for Mn^2+^-free, wild-type and mutant riboswitch to figure out the correlation or anti-correlation between nucleotides of the M_B_ site and other regions, in particular the P1.1 switch helix (Fig. 5). DCCM plots depicted correlated movements between residues belonging to different regions of the riboswitches which were observed throughout 1 μs trajectories of each riboswitch. In DCCM plot, correlation range was from +1 to −1 representing with variation in the color intensity; red-colored contours correspond to positively correlated movements, white-colored regions represent zero correlation or uncorrelated movements and blue-colored contours signify negatively or anti-correlated movements. The characteristic features in the DCCM plots were depicted as boxed regions labelled as 1 to 16 showing effects of Mn^2+^ absence and A41U mutation on the structure and dynamics of the riboswitch. In the case of Mn^2+^-free riboswitch, region 8, and 3 and 5 corresponded to positively correlated and zero correlated motions, respectively (Fig. 5a), whereas the presence of Mn^2+^ ion in the wild-type riboswitch caused the riboswitch to attain mostly positively correlated motions (Fig. 5b). The A41U mutation was demonstrated to affect the atomic motions causing the mutant riboswitch to adopt distinct motions for instance regions 12, 13, 15 and 16 corresponded to zero correlation (Fig. 5c). Moreover, a contrast was observed between Mn^2+^-free and wild-type cases, as regions 1 and 7 in the Mn^2+^-free case depicted positively correlated motions compared to the corresponding region with zero correlated motions. Similarily, a constrast was observed for the case of mutant riboswitch when comparing with the wild-type case; regions 7, and 2, 9, 14 and 11 in the mutant case corresponded to positively and negatively correlated motions, respectively, whereas Mn^2+^-free case had mixed correlations consisting of both positive and negative correlations for the atomic motions corresponding to respective regions 1 and 7, and 2, 4, 6, 9 and 10. Nevertheless, the difference in the DCCM plots was too small to observe the obvious effect of Mn^2+^-absence and A41U mutation on the correlation dynamics among different regions, particularly, between M_B_ site nucleotides (G9, U44, A41, C45, U39 and C40) and nucleotides of P1.1 switch helix (A3, A4, A5, G6, G7, C96, C97, U98, U99, U100, C101 and C102) of the riboswitches, which provoked to perform essential dynamics (ED) or principal component analysis (PCA) in order to evaluate distinct dynamics of the riboswitches.

**Fig. 6:**
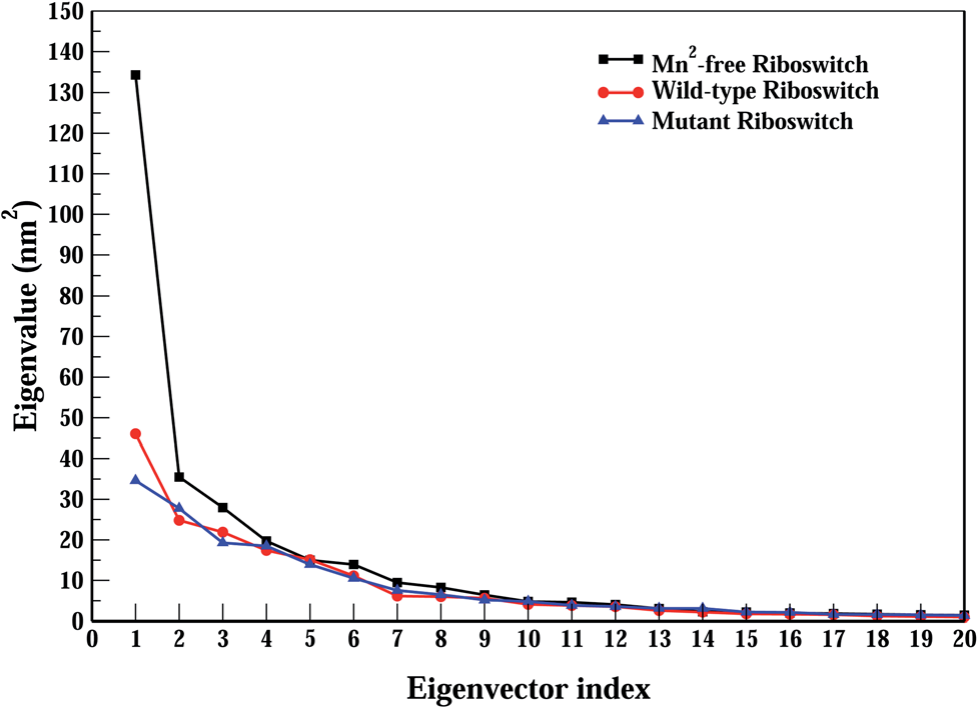
Screen plot depicting first 20 eigenvalues of covariance matrix obtained during principal component analysis of Mn^2+^-free (black), wild-type (red) & mutant (blue) yybP–ykoY riboswitches.

PCA reduces the dimensionality of huge data set and helps to identify significant motions which are incumbent for the biological activity of a molecule.^39^ As the essential motions are extracted from sampled conformations, the method of applying PCA to a simulated trajectory is called essential dynamics (ED).^40–42^ With the removal of roto-translation motions of all atoms as of the nucleic acid, conformations are collected throughout the MD trajectory. Consequently, covariance matrix is set up that upon diagonalization of system's atomic coordinates targets the significant motions of conformation which are accountable for the mass variance in atomic positions as eigenvalues. Eigenvectors (essential modes) are determined by ranking eigenvalues of principal components (PCs) represented by scree plot shown in Fig. 6. Afterward, essential conformational space is determined by projecting the original configuration of the system on the PCs.^39^ Essential subspace comprises a few degree of freedom, in which positional fluctuations occur an-harmonically, therefore, essential degree of freedom points out the motions relevant to the function of biomolecules.^42^ Here, eigenvalues from covariance matrix were constructed from all-atom coordinates of the Mn^2+^-free, the wild-type and the mutant riboswitches which are shown in descending order. Eigenvalues of the Mn^2+^-free case showed steeper decrease than those of the wild-type and the mutant cases, and the wild-type case also showed steeper decrease than the mutant case. Trace of the covariance matrix of the Mn^2+^-free case happened to be 332.71 nm^2^ whereas for the wild-type and mutant cases, the values yielded to be 203.66 and 205.39 nm^2^, respectively. Since the high values of trace of the covariance matrix generally pointed toward more flexible system,^39^ therefore, high value of trace of covariance in case of Mn^2+^-free riboswitch demonstrated high dynamic flexibility due to Mn^2+^ absence in comparison to other two riboswitches, and the mutant case with slightly high trace of covariance matrix was assessed to have slightly more flexible structure compared to the wild-type riboswitch. Moreover, large eigenvalues for the case of Mn^2+^-free riboswitch suggested to be less compact structure compared to the wild-type and the mutant riboswitches, thus giving a more clear description of the volume of Mn^2+^-free and wild-type riboswitches as described earlier by *R*_g_ calculations (see Fig. 3a). The first two eigenvectors were selected for the evaluation of essential dynamics of the riboswitches since they corresponded to the highest eigenvalues demonstrated by visible kink in the scree plot (Fig. 6).

**Fig. 7:**
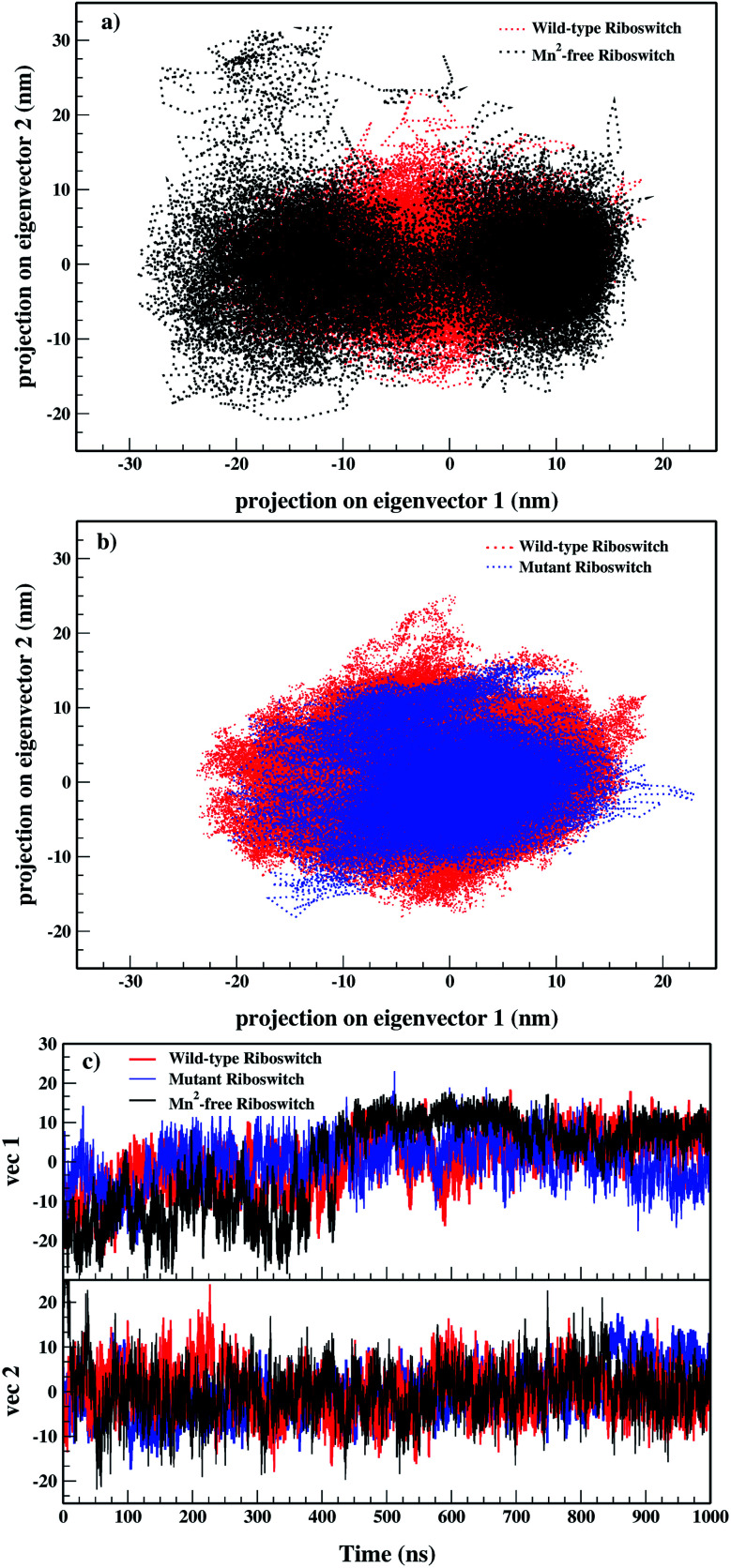
2D projection of simulated trajectories of (a) wild-type (red) & Mn^2+^-free (black) and (b) wild-type (red) & mutant (blue), on initial two eigenvectors. (c) 1D projection of simulated trajectories (1 μs) of Mn^2+^-free, wild-type & mutant yybP-ykoY riboswitches on initial two eigenvectors with respect to time.

**Fig. 8:**
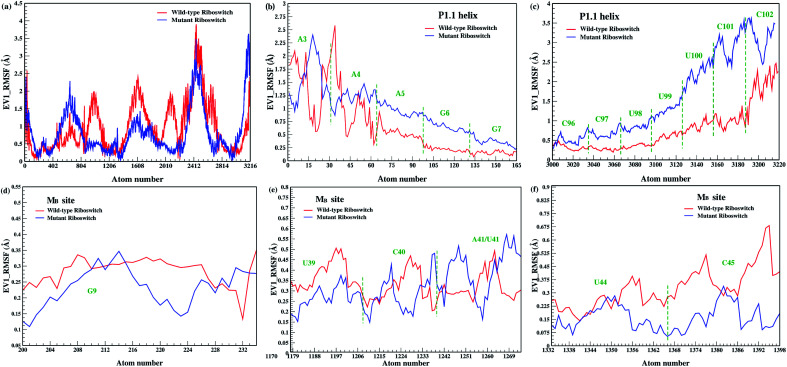
Root mean square fluctuation (RMSF) of the atoms along eigenvector-1 (EV1) during ED analysis of (a) wild-type and mutant riboswitches, (b) and (c) of the P1.1 switch helix and (d)–(f) of M_B_ site residues.

Conformational sampling of Mn^2+^-free, wild-type and mutant riboswitches in the essential subspace was depicted in Fig. 7, illustrating the conformations along the two eigenvectors (EV1 and EV2) projected by all-atoms. It was observed that Mn^2+^-free riboswitch occupied larger and distinct conformational subspace than wild-type riboswitch, however, atomic positions in the Mn^2+^-free case was also overlapping with that of the wild-type case showing some differences in the sampling of Mn^2+^-free and wild-type riboswitches, thus pointing toward the change in the conformational space of the riboswitch due to Mn^2+^ ion (Fig. 7a). On the other hand, the mutant riboswitch occupied smaller conformational subspace than the wild-type riboswitch and atomic positions nearly overlapped with that of the wild-type riboswitch demonstrating that the mutation stabilized the overall structure of riboswitch and also decreased conformational subspace occupied by the mutant riboswitch (Fig. 7b). To further ascertain the mutation effect on the riboswitch dynamics in particular the M_B_ site and the P1.1 switch helix, atomic fluctuations of nucleotides, and dynamic correlation between the M_B_ site and the P1.1 switch helix, accountable for the essential subspace dynamics were analyzed *via* RMSF calculations along EV1 during PCA, since it reduced the functionally irrelevant motions and unmasked functionally significant motions (Fig. 8). The PCA-based RMSF helped to gain further insight into the essential dynamics of the riboswitch that was not achieved earlier by simple RMSF calculations and DCCM analysis (see Fig. 4 and 5).

Fig. 8a showed that the mutation decreased the flexibility of the overall structure of the riboswitch, as low fluctuations were observed in the RMSF profile of the mutant riboswitch thus revealing that the mutation stabilized the overall structure which also correlated the backbone RMSD of the mutant riboswitch showing enhanced structural stability (see Fig. 2a). PCA based RMSF analysis also demonstrated low fluctuations of the M_B_ site nucleotides in the mutant riboswitch such as G9, U39, U44 and C45 except C40 and U41 depicted in Fig. 8d–f. The RMSF profile also revealed that the mutation affected the overall dynamics of the riboswitch but not the flexibility of the M_B_ site nucleotides rather than make the metal site more rigid. On the other hand, all nucleotides (A4, A5, C96, C97, U98, U99, U100, C101 and C102) except A3 in the P1.1 switch helix of the mutant riboswitch showed relatively high fluctuations depicted in Fig. 8b and c, demonstrating that the mutation increased the helix flexibility. This finding complemented an experimental study reporting Mn^2+^ binding at the M_B_ site that stabilized P1.1 switch helix in yybP-ykoY riboswitch, and this stabilizing effect was lost upon Mn^2+^ sensing adenosine mutation.^14^

**Fig. 9:**
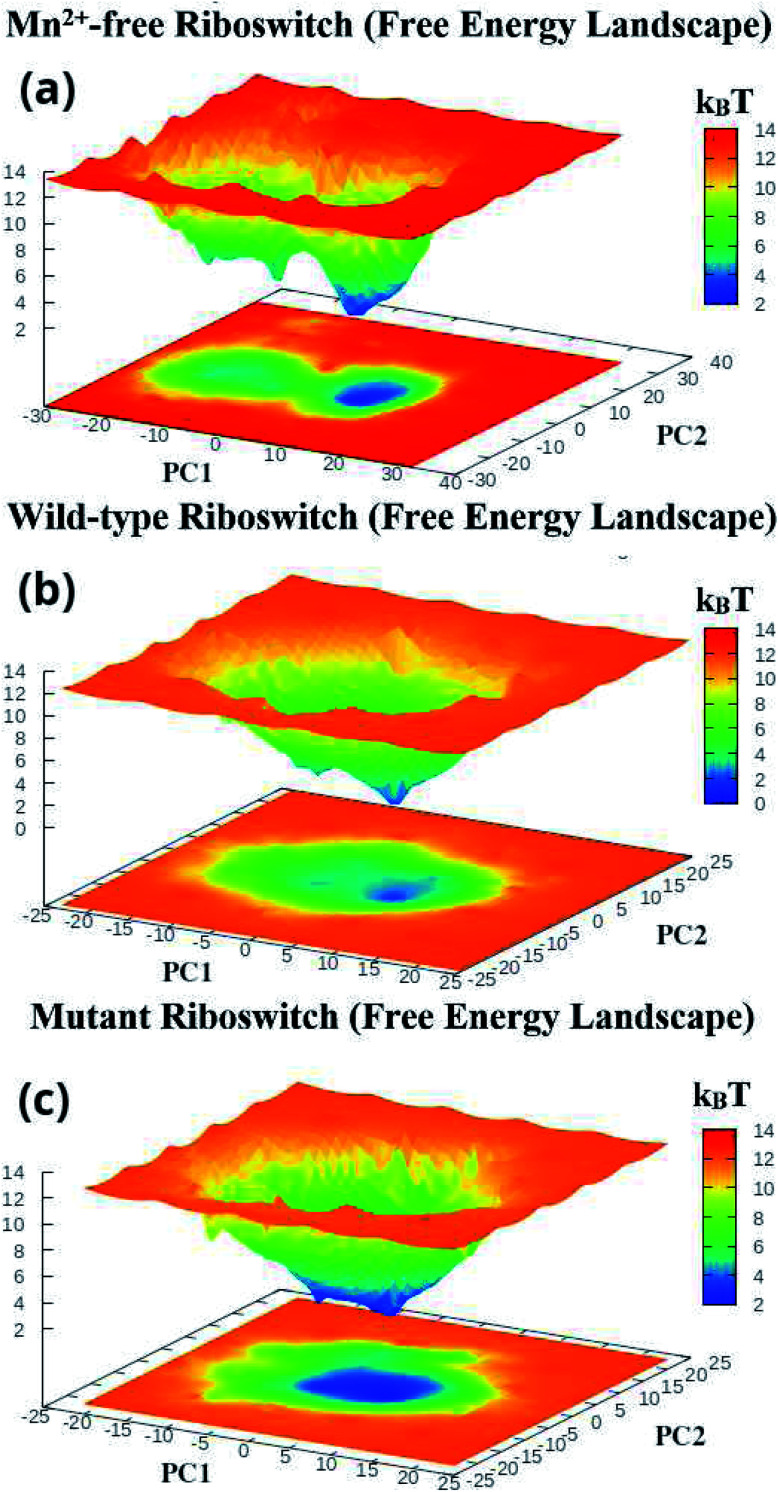
Gibbs free energy landscapes of PC1 & PC2 (initial two principal components) of (a) projection of simulated trajectories of (a) Mn^2+^-free, (b) wild-type and (c) mutant riboswitches, obtained during (1 μs) MD simulations.

The first two principal components, PC1 and PC2 of each Mn^2+^-free, wild-type and mutant riboswitch were employed to obtain information of the global minimum-energy (stable) conformations responsible for the essential dynamics, which were depicted in the form of Gibbs free energy landscapes (FEL) shown in Fig. 9. The lowest energy states or stable conformations represented by blue colored maps whereas red colored maps corresponded to high-energy conformations. Each riboswitch produced different FEL pattern; the Mn^2+^-free case showed two free energy basins (valleys) with pointed and flat ends, which were less broad than the single energy basin observed in the wild-type case (Fig. 9a and b). The minima with flat ends corresponded to clusters of stable conformations, the minima with pointed end represented a stable conformation, and energy barriers were large between basins unlike between the minima in basins. Since narrow energy valleys indicated small number of conformations, the Mn^2+^-free case yielded less number of stable conformations in comparison to the wild-type riboswitch. FEL of the mutant riboswitch depicted broader minima with little energy barrier than that of the wild-type riboswitch; minima with flat ends indicated clustering of the stable or low energy conformations, and a canonical end suggested the presence of a stable conformation. In the case of mutant riboswitch, large number of stable conformations were produced than the wild-type riboswitch as reflected by FEL pattern (Fig. 9c). The stable conformations indicated by the minima were responsible for essential dynamics in the riboswitch, and as the number of stable conformations decreases flexibility of the system increases and *vice versa*. Consequently, FEL pattern served to figure out the effect of the mutation and Mn^2+^ absence on the sampled essential subspace.

**Fig. 10:**
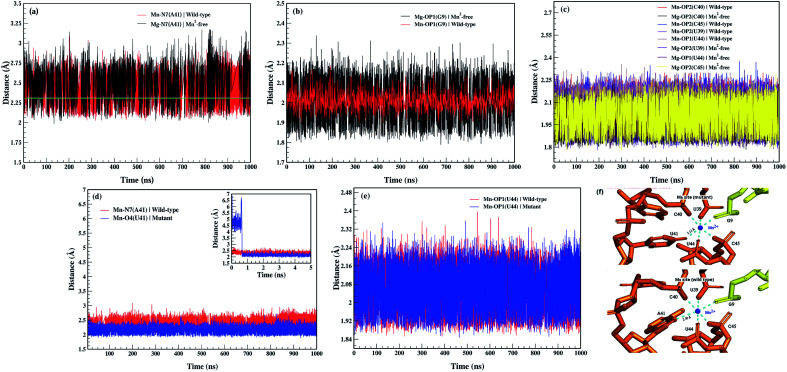
(a) Distance between N7_A41_ and Mg^2+^, and N7_A41_ and Mn^2+^ of Mn^2+^-free and wild-type riboswitches, respectively, (b) distance between OP1_G9_ and Mg^2+^, and OP1_G9_ and Mn^2+^ of Mn^2+^-free and wild-type riboswitches, respectively, (c) distance between Mg^2+^ and OP1_U44_, OP2_U39_, OP2_C45_, OP2_C40_ of Mn^2+^-free, and Mn^2+^ and OP1_U44_, OP2_U39_, OP2_C45_, OP2_C40_ of wild-type riboswitch, (d) distance between O4_U41_ and Mn^2+^ of mutant riboswitch, and N7_A41_ and Mn^2+^ of wild-type riboswitch, (e) distance between OP1_U44_ and Mn^2+^ of mutant riboswitch, and OP1_U44_ and Mn^2+^ of wild-type riboswitch, (f) M_B_ site of mutant and wild-type riboswitches.

The coordination sphere of the metal binding M_B_ site was also analyzed by evaluating dynamics of its geometric configuration in terms of bond lengths and bond angles. Fig. 10 illustrated time evolution coordination distances between the metal ion and the coordinating atoms of the nucleotides in the Mn^2+^-free, the wild-type and the mutant riboswitches. Based on the distance plots, the averaged coordination distances were computed for the metal-nucleotide atomic pairs in each riboswitch and listed in Table 1. In case of Mn^2+^-free riboswitch, distances between Mg^2+^ and the M_B_ site residues were averaged to be less than 2 Å throughout the simulation time except Mg–N7_A41_ distance (2.31 ± 0.11 Å) that was same as the Mn–N7_A41_ distance (2.30 ± 0.09 Å) in the wild-type riboswitch, thus demonstrating the similar structure behavior of these two metal ions. Nevertheless, based on the distance plots, the average coordination distances were computed between the metal ions and nucleotides of the M_B_ site in each riboswitch. In case of Mn^2+^-free riboswitch, distances between Mg^2+^ ion and the M_B_ site residues were averaged to be less than 2 Å throughout the simulation time except the Mg–N7_A41_ distance (2.31 ± 0.11 Å) that was same as the Mn–N7_A41_ distance (2.30 ± 0.09 Å) in the wild-type riboswitch. However, the amplitude of these two distance plots had a characteristic constrast as the distance in Mn^2+^-free riboswitch was slightly larger than the wild-type riboswitch (Fig. 10a), reflecting less stability of the M_B_ site in the absence of essential Mn^2+^ ion. Likewise, a relatively large amplitude was observed for Mg–OP1_G9_ distance (1.96 ± 0.05 Å) compared to Mn–OP1_G9_ distance (2.02 ± 0.05 Å) indicating less stability of the metal coordination site even though the distance in the Mn^2+^-free riboswitch was low than the wild-type case (Fig. 10b). Other metal-nucleotide distances in Mn^2+^-free riboswitch were also smaller than those of the wild-type riboswitch but the Mg–nucleotide distances had similar amplitudes (Fig. 10c), suggesting its rigidity than those in the wild-type riboswitch. Based on the distance plots, it was evaluated as the Mg^2+^ ion unlike Mn^2+^ ion showed constant variations while making interactions with the M_B_ site nucleotides, whereas Mn^2+^ being an essential constituent of the riboswitch as of the wild-type, forms dynamically stable metal coordination site which could be attributed to the function of the Mn^2+^-sensing riboswitches. The rigidity of the metal-nucleotide distances in Mn^2+^-free riboswitch also corroborated the RMSF data, demonstrating structural flexibility of the riboswitch (see Fig. 4d–f), however, irrespective of very similar RMSD values for the M_B_ site nucleotides in Mn^2+^-free and wild-type riboswitches. The average distances between Mn^2+^ and M_B_ site residues in the wild-type riboswitch were very close as that of the mutant riboswitch as listed in Table 1, except Mn–N7_A41_ distance (2.30 ± 0.09 Å) in the wild-type that was larger than Mn–O4_U41_ distance (2.12 ± 0.09 Å) in the mutant form shown in Fig. 10d, in which U41 nucleotide was not having N7 atom. This indicated that the mutation led to shorter and rigid contacts between Mn^2+^ and M_B_ site residues which also correlated the PCA results signifying that the flexibility of M_B_ site decreases with mutation (see Fig. 8d–f). Fig. 10d depicted a fragile coordination distance of Mn^2+^ with O4 atom of uracil in the mutant riboswitch that was formed at the beginning of the simulation time as deduced from the distance plot for the first 5 ns, which upon equilibration at ∼1 ns got decreased thus causing the coordination shell to be stabilized, and this finding was found to be correlating with an experimental study^11^ (Fig. 10f). According to the experimental study, the A41U mutation led Mn^2+^ coordination scheme to adopt certain changes in such a way that the coordination scheme of M_A_ site nucleotides and G10, U39, C40, and C45 of M_B_ site remained unaffected, whereas the backbone phosphate atoms of U44 was shifted away and replaced by a water molecule as resolved by 2.2 Å electron density.^11^ Conversely, the simulation study showed that the contact between OP1 atom of U44 nucleotide and Mn^2+^ remained stable and was not substituted by water as demonstrated by the distance plots illustrated in Fig. 10e and f.

**Table 1:** Bond distance of the metal ions with the M_B_ site nucleotides in the Mn^2+^-free, wild-type and mutant riboswitches

Bond distance	MD simulation (Å)	X-ray (Å)^11^
**Mn** ^**2+**^ **-free riboswitch**		
Mg–OP1_G9_	1.96 ± 0.05 Å	—
Mg–OP1_U44_	1.95 ± 0.05 Å	—
Mg–N7_A41_	2.31 ± 0.11 Å	—
Mg–OP2_C45_	1.97 ± 0.05 Å	—
Mg–OP2_U39_	1.95 ± 0.05 Å	—
Mg–OP2_C40_	1.95 ± 0.05 Å	—

**Wild-type riboswitch**		
Mn–OP1_G9_	2.02 ± 0.05 Å	2.21
Mn–OP1_U44_	2.02 ± 0.05 Å	2.23
Mn–N7_A41_	2.30 ± 0.09 Å	2.26
Mn–OP2_C45_	2.02 ± 0.04 Å	2.27
Mn–OP2_U39_	2.02 ± 0.05 Å	2.30
Mn–OP2_C40_	2.01 ± 0.05 Å	2.33

**Mutant riboswitch**		
Mn–OP1_G9_	2.02 ± 0.05 Å	—
Mn–OP1_U44_	2.02 ± 0.05 Å	—
Mn–O4_U41_	2.12 ± 0.09 Å	—
Mn–OP2_C45_	2.02 ± 0.05 Å	—
Mn–OP2_U39_	2.01 ± 0.05 Å	—
Mn–OP2_C40_	2.02 ± 0.05 Å	—

**Fig. 11:**
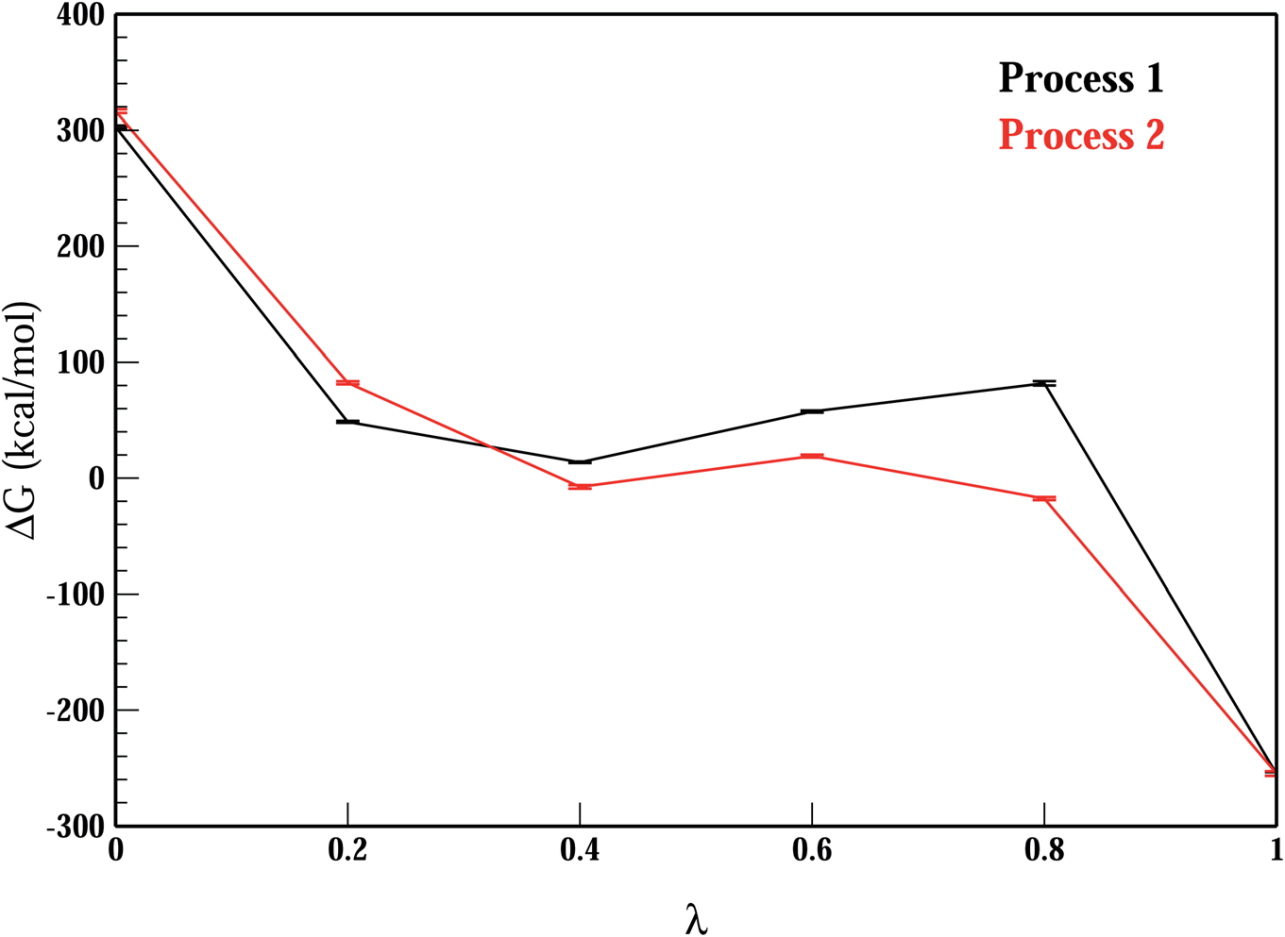
Relative binding free-energy between mutant and wild-type riboswitches as a function of *λ* (coupling parameter) employing thermodynamic integration approach. Process 1 (and 2) outlines free-energy difference for the conversion of wild-type system into mutant system with Mn^2+^ (and without Mn^2+^) at binding site M_B_.

After having analyzed the effects of Mn^2+^ absence and the mutation in terms of structure and dynamics of yybP–ykoY riboswitch, and in particular, the flexibility (stability) of the P1.1 switch helix and the M_B_ site, the A41U mutation effect was quantified as the binding free energy of the mutant riboswitch. The free energy was estimated with respect to the Mn^2+^ ion bound to the wild-type and the mutant riboswitches through relative binding free energy difference (ΔΔ*G*_bind_) *via* free energy simulations based on TI approach,^43^ in order to evaluate the relative stability of the two Mn^2+^-bound riboswitches. Fig. 11 illustrated the plot of relative free energy for each interval of *λ* that is between adjacent Hamiltonian for both riboswitches, where process 1 illustrated binding free energies in the presence of Mn^2+^, computed as a function of coupling parameter, *λ*, whereas binding energy calculation (without Mn^2+^) was carried out in process 2 to obtain useful information employing non-physical thermodynamic cycle (Scheme 1) for the relative binding free energy difference based on the following relation,

2 ΔΔ*G*_bind_ = Δ*G*^B^_Bind_ − Δ*G*^A^_Bind_ = Δ*G*^A⃑B^_1_ − Δ*G*^A⃑B^_2_

Table 2 listed the free energies computed during process 1 (Δ*G*^A⃑B^_1_) and process 2 (Δ*G*^A⃑B^_2_), and the relative binding free energy difference (ΔΔ*G*_bind_) revealed that the mutant riboswitch was more stable than the wild-type riboswitch with relative binding energy difference of 23.75 kcal mol^−1^. The increase in the binding free energy for the mutant riboswitch was attributed to the more rigid contact between O4_U41_ and Mn^2+^ compared to the Mn–N7_A41_ coordination bond in the wild-type riboswitch (Table 1 and Fig. 10). Based on the free energy data, it was obvious that the A41U mutation stabilizes A41U yybP–ykoY riboswitch of *L. lactis*.

**Table 2:** Free energies in kcal mol^−1^ acquired from free energy simulations employing thermodynamic interaction (TI) method

Process	Free energy (kcal mol^−1^)
Δ*G*^A→B^_1_ (process 1)	45.09 ± 0.09
Δ*G*^A→B^_2_ (process 1)	21.34 ± 0.04
ΔΔ*G*_bind_	23.75 ± 0.05

## 4 Conclusion

Based on the simulation data, the presence of Mn^2+^ ion was signified as the ion was essentially required for the proper functioning of yybP-ykoY riboswitch of *L. lactis*. For this purposes, MD simulations were applied to the metal-sensing riboswitches employing the most widely used ff99bsc0χOL3 AMBER force field to model and describe RNA topology despite having several reports that the force field was unable to reproduce some tetranucleotides and tetraloops for the conformational features and NMR experimental data, and therefore, a number refinements and optimizations of the force field were suggested.^44^ Nevertheless, the data obtained from the simulation was found to be in good agreement with the experimental data reported so far. Furthermore, the application of non-bonded parameter for the manganese and magnesium ions was also found to be compatible as the structural and dynamical properties of the metal-binding sites reproduced well when compared with experimental data based on X-ray crystallography.^11^ The absence of Mn^2+^ ion at the M_B_ site of the Mn^2+^-free riboswitch was evaluated as the structurally similar Mg^2+^ ion from the solution attempted to mimic the functional role of the essentially important Mn^2+^ ion in the riboswitch but the dynamical properties of the two ions were reported to be distinct which were suggested to be associated to their different functional role in the biological molecules. The simulation results thus yielded that the presence of Mn^2+^ ion was essential for the Mn^2+^-sensing riboswitch which in the absence of Mn^2+^ ion undergo unfavourable structural and dynamical modifications. Moreover, the effect of the A41U mutation in the riboswitch was examined and it was found that the mutation affected not only the overall riboswitch structure but also the metal-binding site and the P1.1 switch helix, which undergo structural and conformational modifications due to mutation. The evaluation of the structure and dynamics related to the metal-binding site and the P1.1 switch helix obtained from the simulations was expected to serve further in the future studies with focus on deciphering the functional role of the Mn^2+^-sensing riboswitch which in turn could be helpful in unravelling how the metal-sensing riboswitch could be considered for therapeutic purposes.

## Conflicts of interest

There are no conflicts to declare.

## Acknowledgments

## Supplementary Material
